# Myocardial oedema in an 8-year-old Chinese boy with Idiopathic systemic capillary leak syndrome

**DOI:** 10.1186/s12887-019-1401-2

**Published:** 2019-01-21

**Authors:** Karen Ka Yan Leung, Jaime Sou Rosa Duque, Kwong-Man Yu, Kai-Ning Cheong, Patrick Chun-Yin Chong, Marco Hok-Kung Ho, Pak-Cheong Chow

**Affiliations:** 1 Department of Paediatrics and Adolescent Medicine, Queen Mary Hospital, Room 115, New Clinical Building, 102 Pokfulam Road, Pokfulam, Hong Kong; 2 Department of Paediatric Cardiology, Queen Mary Hospital, Room 322, New Clinical Building, 102 Pokfulam Road, Pokfulam, Hong Kong

**Keywords:** Systemic capillary leak syndrome, Clarkson’s disease, Paediatrics, Shock, Myocardial oedema, Extracorporeal membrane oxygenation, ECMO

## Abstract

**Background:**

Idiopathic systemic capillary leak syndrome (ISCLS) is rare, and there has been about 32 cases reported in children worldwide since this disorder was first described in 1960. Clinical guidelines on the management approach stemming from robust scientific evidence are lacking. This case report presents the first reported paediatric case of severe ISCLS with significant myocardial oedema and emphasizes this disease’s impact on a child’s cardiac function.

**Case presentation:**

A Chinese boy had his first attack of severe hypovolaemic shock that responded to fluid resuscitation when he was 6 years of age. His second attack developed at 8 years of age. He was then transferred to our cardiac unit for refractory hypotensive shock. The patient’s echocardiogram revealed ventricular wall thickening with significant cardiac dysfunction requiring extracorporeal membrane oxygenation support. Subsequently, he made a full recovery, including his myocardial wall thickness and function. The echocardiographic findings suggested myocardial oedema that was transient in nature. Clinical and laboratory investigation from both episodes were compatible with ISCLS.

**Conclusion:**

ISCLS is rare, and therefore there is only a limited understanding on the pathophysiology of this disorder. The current treatment approach is based on a few case reports and series. During the acute phase, optimal supportive management is paramount. Our case highlights the importance of early recognition and consideration for extracorporeal membrane oxygenation support in patients with a life-threatening presentation, as it was lifesaving for this child who suffered myocardial oedema and ventricular dysfunction.

## Background

Idiopathic systemic capillary leak syndrome (ISCLS), also known as Clarkson’s disease, was first described by Dr. Bayard Clarkson in 1960 [1]. It appears to be a rare disorder characterized by changes in capillary permeability resulting in a sudden massive shift of plasma from the circulatory system into the interstitial space. The original proposed classic triad consisted of severe hypotension, hypoalbuminaemia and haemoconcentration [1, 2]. In children, signs of tissue hypoperfusion may be more evident than hypotension [3]. Herein we described the first paediatric case of life-threatening ISCLS resulting in myocardial oedema with circulatory failure. This patient required extracorporeal membrane oxygenation (ECMO) support and subsequently made a full recovery.

### Case presentation

A Chinese boy with a medical history of eczema and obesity presented with two episodes of anasarca and hypovolemic shock.

**Fig. 1:**
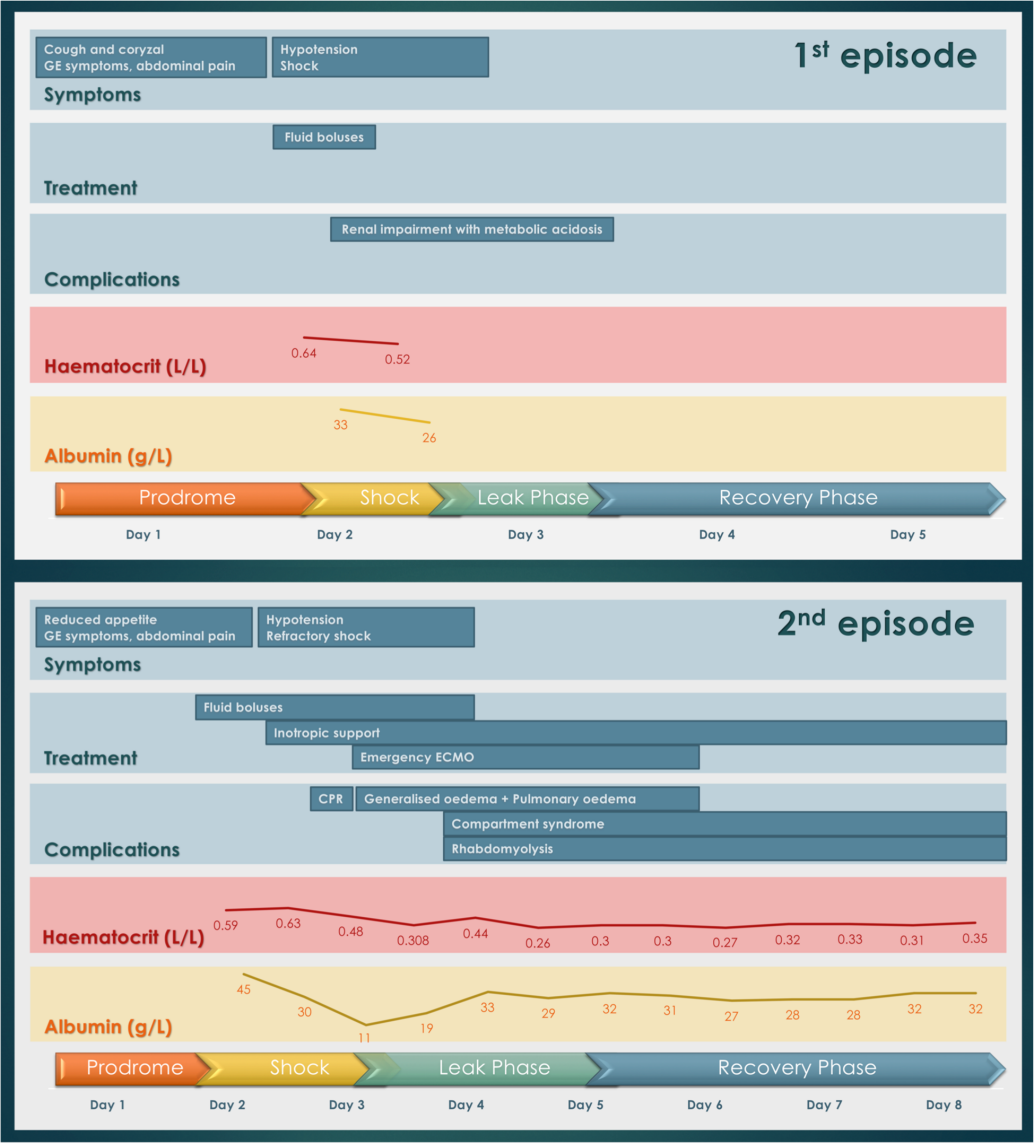
A summary of clinical findings and investigations of the first and second episodes of shock. Both episode of shock started with a viral prodrome and progressed to hypotension and shock. The laboratory findings also revealed haemoconcentration and hypoalbuminaemia. Complications developed in the second episode included pulmonary oedema, compartment syndrome and rhabdomyolysis

When the patient was six years old, he had coryzal symptoms for two days and a one-day history of vomiting, diarrhoea and generalised abdominal pain. His blood pressure was 85/66 mmHg and heart rate was 144 beats per minute (bpm) upon presentation at another local hospital. This progressed to hypovolaemic shock requiring admission to the paediatric intensive care unit for fluid resuscitation. Laboratory investigation showed haemoconcentration, hypoalbuminemia and renal impairment with metabolic acidosis (Fig. 1). Echocardiogram revealed a thickened left ventricle. Blood culture yielded coagulase-negative *Staphylococci*, which was deemed a skin contaminant. He was treated with fluid resuscitation and a seven-day empiric course of ceftriaxone. The patient’s renal function normalised after fluid replacement and he was discharged after one week. An echocardiogram was repeated a month later, which showed normal ventricular wall thickness, structure and function.

The patient was well afterwards until he was eight years old. He presented with vomiting, diarrhoea, abdominal pain and a low-grade fever for one day. Again, he was admitted to another local hospital. The child became lethargic, hypotensive (76/52 mmHg), tachycardic (141 bpm), with physical signs of poor perfusion. Within 8 h of admission, a total of 3500 ml (70 mL/kg) of normal saline boluses were given but there were only transient periods of improvement and the blood pressure remained low overall. Laboratory investigation again showed haemoconcentration, hypoalbuminemia, impaired renal function and metabolic acidosis (Fig. 1). Echocardiogram from the referring hospital showed a thickened left ventricle; the interventricular septum was 11.9 mm (Z-score + 13.34) and free wall was 14.2 mm (Z-score + 16.22).

His blood pressure remained unstable despite additional boluses of 3500 ml (70 mL/kg) of normal saline in total over the next 12 h. Multiple inotropic medications including dopamine, dobutamine, noradrenaline and stress dose hydrocortisone were started. He was transferred to our hospital, a tertiary referral centre, for consideration of ECMO within 24 h of admission.

**Fig. 2:**
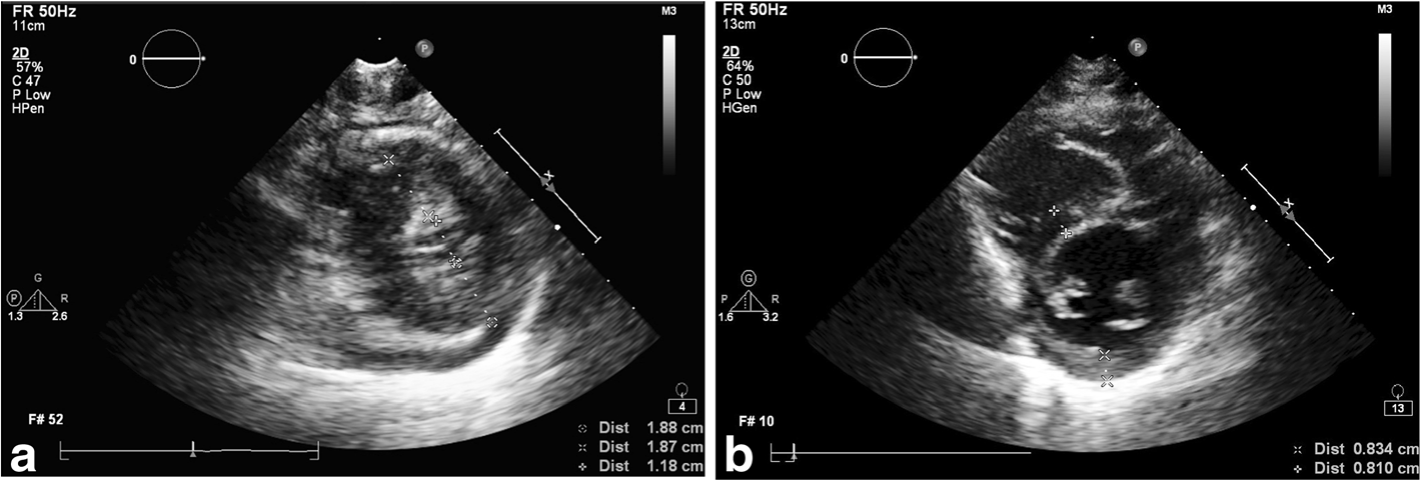
**a.** Echocardiogram after cardiac arrest during the second presentation. Left ventricular wall thickening with underfilled left ventricle. **b.** Echocardiogram 7 days after the second presentation. Left ventricular wall thickening normalised

During the transfer, he was given a total of 11 intravenous boluses (total 21 mL) of 0.1 mg/mL adrenaline due to persistent shock. He developed pulseless electrical activities shortly after arrival to our cardiac intensive care unit and recovered after two minutes of cardiopulmonary resuscitation. Upon return of spontaneous circulation, his blood pressure was 44/37 mmHg and his heart rate was 185 bpm. Post-resuscitation echocardiogram showed poor systolic function of both ventricles with the left ventricular internal dimension at end-diastole (LVIDd) of 18.3 mm (z-score − 8.65); the intraventricular septum at end-diastole (IVSd) was 18.3 mm (z-score + 4.61); left ventricular posterior wall at end-diastole (LVPWd) was 13.4 mm (z-score + 3.95)**;** left ventricle fractional shortening (LVFS) was 18.3%; tricuspid annular plane systolic excursion (TAPSE) was 14.3 mm (z-score − 3.60) and the left ventricular mass index was 70 g/m^2^ (Fig. 2a).

Emergency central veno-arterial ECMO was immediately initiated via cannulation of the right atrium and ascending aorta, and another 5200 ml (100 mL/kg) of fluid and blood products were given over the first 12 h after ECMO was started to maintain his intravascular volume. An optimal ECMO flow was achieved at 3 L/min with a cardiac index of 2.2 L/min/m^2^ afterwards. His nasopharynx aspirate was positive for parainfluenza virus type 2 and stool culture was positive for *Salmonella* group B. Blood culture and urine culture were negative and there was no detectable urinary protein. The patient had a mildly low immunoglobulin (Ig) G level of 687 (724–1380) mg/dL, but normal IgA and IgM. Protein electrophoresis and urine immunofixation showed no monoclonal antibody peak.

The patient was very oedematous and developed compartment syndrome involving both lower limbs that required emergency fasciotomy. He also developed rhabdomyolysis with deranged renal function and pulmonary oedema 24 h after ECMO was initiated. Echocardiogram repeated 12 h after ECMO insertion showed reduced thickening of his left ventricular wall. The patient’s cardiac function, renal function and perfusion subsequently improved. Inotropic and ECMO support was weaned off three days after initiation. Echocardiogram four days after termination of ECMO support showed improvement of the left ventricular dimension and wall thickness and cardiac function: LVIDd was 33.4 mm (z-score − 2.63); IVSd was 6.95 mm (z-score 0.09); LVPWd was 6.62 mm (z-score 0.28); LVFS was 32.6% and the left ventricular mass was 39 g/m^2^ (Fig. 2b).

As the patient’s clinical and laboratory findings were all suggestive of ISCLS after discussion with an immunologist, montelukast 5 mg daily was started as prophylactic treatment at three weeks, and he received his first monthly immunoglobulin infusion (1 g/kg) prophylaxis two months after the initial presentation. At his subsequent follow-up appointment, he still had residual peroneal neuropathy. Echocardiogram 11 weeks later showed normal left ventricular wall thickness and a full recovery of his biventricular function. He has remained asymptomatic so far for more than 12 months at his most recent clinic follow-up.

### Discussion and conclusions

ISCLS is a rare, life-threatening disease with a peak occurrence during middle-age adulthood. There has been only a few case reports and series involving children. As such, we reviewed the literature focusing on cardiac involvement, as well as the acute and prophylactic management of ISCLS in children.

Approximately 500 adults and 32 paediatric cases of ISCLS have been described [2–19]. Most of these cases occurred in Western countries. Our patient is the first reported case of ISCLS in the Chinese population.

### Cardiac involvement in systemic capillary syndrome

Cardiac involvement in ISCLS is relatively uncommon. Findings reported in the literature included pericardial effusion [4], ventricular hypertrophy [9], left ventricular dysfunction [20], myocardial stunning [11] and myocardial oedema [21–24]. Echocardiogram showed ventricular wall thickening suggestive of myocardial oedema, cardiac dysfunction and ventricle was underfilled due to distribution of intravascular volume by capillary leak. There has been only four adult ISCLS cases with myocardia oedema (retrospective inference by the echocardiographic findings of ventricular wall thickening or biopsy findings) (Table 1) [21–24]. For the three life-threatening adult cases with myocardial oedema, biopsies of the heart demonstrated diffuse oedema without inflammatory infiltrates or myocyte necrosis [21, 23]. One patient required central extracorporeal life support to bridge to recovery [21], while the two other patients died [23, 24]. For the one patient who survived, the myocardial oedema was transient and reversible. Myocardial oedema, together with severe ventricular dysfunction, may be a complication of the more severe, life-threatening attacks (Table 1). When these occur, ECMO support should be considered early as it can be lifesaving.

**Table 1:** Cases of idiopathic systemic capillary leak syndrome with features suggestive of myocardial oedema

	Age (Years)	Sex	Clinical course	ECHO	Histopathology	ECMO support	Outcome
Claessens et al 1998 [21]	53	M	Prodromal symptoms of chills, fever and cough, presented with shock, oedema	Global myocardial wall thickening Normal contractility Mild and non-compressive effusion	Not done	No	ECHO normalised within 3 days, alive
			Prodromal symptoms of cough, vomiting and shock	Myocardial wall enlargement Normal systolic function	Not done	No	ECHO normalised within 3 days, alive
Juthier et al 2012 [20]	41	M	Known history of SCLS, presented with chest pain associated with ECG changes of ST segment elevation Developed sudden circulatory collapse	Left ventricular wall thickness was mildly increased Severely reduced right and left ventricle systolic function	Diffuse interstitial oedema. No inflammatory infiltrate and myocyte necrosis.	Yes	ECHO normalised in 17 days, alive
Ertel et al 2015 [22]	54	M	Known ISCLS, presented with prodromal symptoms of upper respiratory tract infection, developed shock, multiple organ failure	Not available	(Post mortem) Diffuse oedema within the myocardial wall. Lace-like fibrosis of the interstitial space in the myocardium, consisted with sustained and/or recurrent, intermittent episodes of myocardial oedema No myocyte necrosis or lymphocytic infiltrates	No	Died
Zancanaro et al 2015 [23]	49	M	Known ISCLS, presented after 2 days of sustained physical exertion with progressive oedema and hypotension, died after 15 hours with multiple organ failure and sudden cardiac arrest	Not available	(Post mortem) Myocardium with remarkable interstitial oedema with evidence of acute myocardial ischemia	No	Died
Our case	6	M	Prodromal symptoms of gastroenteritis and presented with shock	Ventricular wall thickening suggestive of myocardial oedema	Not done	No	ECHO normalised within 1 month, alive
	8		Prodromal symptoms of gastroenteritis and refractory shock	Ventricular wall thickening suggestive of myocardial oedema Poor systolic and diastolic function	Not done	Yes	ECHO normalised within 1 month, alive

Echocardiography can be used to detect ventricular wall thickening, a finding suggestive of myocardial oedema. Although the cardiac magnetic resonance imaging is the actual modality of choice to definitively confirm this pathological state, at times its accessibility is limited especially when a patient is in an unstable and critical condition.

### Pathophysiology of systemic capillary leak syndrome

The exact pathophysiology of this syndrome is not completely known. It has been postulated that a release of yet to be identified substances in the plasma may lead to a sudden increase in capillary permeability, resulting in massive extravasation of proteins and fluid shift from the intravascular compartment to the extravascular space [1]. Studies have found that patients with acute ISCLS attacks had significantly higher vascular endothelial growth factor and angiopoietin 2 levels than healthy controls, causing disruption of endothelial adherens junctions and cell retraction, leading to an increase in capillary permeability [25]. Inflammatory cytokines (e.g., interleukin (IL) IL-1β, IL-2, IL-6, IL-8, IL-12, IL-17)) [4, 18, 26], leukotrienes [27] and tumour necrosis factor-α (TNF-α) [4] can similarly increase capillary permeability. These factors may play a major role in this disorder as they were found to be elevated in sera samples from patients suffering from acute ISCLS attacks. Monoclonal gammopathy of undetermined significance was found in 85–95% of adult cases [28], but this has not been a prevailing feature in paediatric cases, suggesting that the underlying molecular aetiology of this disorder may be different between adults and children.

### Acute treatment

The first episode of ISCLS usually remains undiagnosed as the presentation is similar to many other diseases such as sepsis and angioedema. If ISCLS is suspected, a secure vascular access is essential for preparing rapid and repeated fluid infusion. There has been no data to clearly demonstrate superiority of one specific type of fluid over another. Overly aggressive fluid rehydration can potentially lead to complication such as compartment syndrome during the leak phase and pulmonary oedema in the recovery phase. Diuretic therapy can be useful as the patient enters the recovery phase [29]. Refractory shock due to ISCLS requiring ECMO support is rare.

Empiric trials of pharmacological agents for the acute management of ISCLS had included intravenous immunoglobulin (IVIG), corticosteroids, theophylline, terbutaline and TNF-α antagonists (e.g., infliximab) [8]. IVIG had seemingly been the most effective in adults with ISCLS [30]. The rationale for the use of IVIG is based on its anti-inflammatory and immunomodulatory properties, anti-idiotypic effect against autoantibodies, and inhibition of complement-mediated damage, all of which should result in less vascular permeability [31, 32]. Both theophylline and terbutaline increase cyclic adenosine monophosphate levels within endothelial cells, which should reduce the capillary leak [8, 33].

The dose and duration of treatment varied between different reports in children, and the effectiveness of these therapies during the acute phase is controversial [7–9, 13]. We did not start any pharmacological agents for our patient during the acute phase as this child was already improving significantly from supportive treatment alone.

### Prophylactic treatment

Prophylactic treatment previously reported in children included IVIG, terbutaline, theophylline, montelukast and gingko biloba. The doses varied and there were no established optimal duration of treatment in the literature.

Druey and Greipp recommended oral terbutaline and theophylline as the first-line prophylactic therapy for adults [31, 34]. However, theophylline has a high adverse-effect profile and narrow therapeutic window in children. For these reasons, we avoided the use of theophylline for our patient.

IVIG appears to be the most promising prophylactic treatment in children. It had been well tolerated with minimal side effects. The doses used varied from 1 to 2 g/kg, infused once a month. Subcutaneous immunoglobulin can be considered for patients who experience significant adverse effects or prefer the convenience of home infusion [4, 35].

Montelukast, a leukotriene receptor antagonist, can theoretically inhibit leukotriene-induced capillary leakage [36]. The majority of reported adverse effects were mild (e.g., headache, ear infection, nausea and abdominal pain) [37]; thus, it may be a better alternative for children. The use of montelukast had been reported in two children in the literature. One patient experienced reduced frequency and severity of attacks [17] while the other suffered one episode of life-threatening relapse of ISCLS [4]. Since montelukast is well tolerated in children and there is anecdotal evidence suggestive of potential benefits, it may be considered as a prophylactic option.

Our patient is the first known case who has been started on IVIG and montelukast. We decided on this combination due to the observed effectiveness and minimal side effect profile as aforementioned. So far, he has been symptom free for 12 months.

## Conclusion

Though ISCLS is rare in children, this disorder should be suspected when a patient presents with a rapid development of hypotension or signs of tissue hypoperfusion, haemoconcentration and hypoalbuminemia [1–3]. Myocardial oedema and ventricular dysfunction in ISCLS may represent a more fulminant course and could be potentially life-threatening. We suggest early performance of echocardiogram on patients with ISCLS to identify myocardial involvement and consider transfer to a specialised centre with ECMO support if available when there is evidence of myocardial oedema.

## Abbreviations

Bpm: Beats per minute
CK: Creatinine kinase
CPR: Cardiopulmonary resuscitation
ECHO: Echocardiogram
ECMO: Extracorporeal membrane oxygenation
Ig: Immunoglobulin
IL: Interleukin
ISCLS: Idiopathic systemic capillary leak syndrome
IVIG: Intravenous immunoglobulin
IVS: Intraventricular septum
IVSd: Intraventricular septum at end-diastolic
IVSs: Intraventricular septum at end-systolic
LVFS: Left ventricle fractional shortening
LVIDd: Left ventricular internal dimension at end-diastole
LVIDs: Left ventricular internal dimension at end-systole
LVPW: Left ventricle posterior wall
LVPWd: Left ventricular posterior wall at end-diastole
LVPWs: Left ventricular posterior wall at end-systolic
TAPSE: Tricuspid Annular Plane Systolic Excursion
TNF-α: Tumour necrosis factor-α
